# A Convenient Ultrasound-Promoted Synthesis of Some New Thiazole Derivatives Bearing a Coumarin Nucleus and Their Cytotoxic Activity

**DOI:** 10.3390/molecules17089335

**Published:** 2012-08-03

**Authors:** Sobhi M. Gomha, Khaled D. Khalil

**Affiliations:** 1 Department of Chemistry, Faculty of Science, University of Cairo, Giza 12613, Egypt; 2 Chemistry Department, Faculty of Science, University of Kuwait, P.O. Box 5969, Safat 13060, Kuwait; Email: khd.khalil@yahoo.com

**Keywords:** thiosemicarbazides, thiazoles, hydrazonoyl halides, ultra-sound irradiation, cytotoxic activity

## Abstract

Successful implementation of ultrasound irradiation for the rapid synthesis of a novel series of 3-[1-(4-substituted-5-(aryldiazenyl)thiazol-2-yl)hydrazono)ethyl]-2*H*-chromen-2-ones **5a**–**h**, *via* reactions of 2-(1-(2-oxo-2*H*-chromen-3-yl)ethylidene) thiosemicarbazide (**2**) and the hydrazonoyl halides **3**(**4**), was demonstrated. Also, a new series of 5-arylidene-2-(2-(1-(2-oxo-2*H*-chromen-3-yl)ethylidene)hydrazinyl)thiazol-4(5*H*)-ones **10a**–**d** were synthesized from reaction of **2** with chloroacetic acid and different aldehydes. Moreover, reaction of 2-cyano-*N'*-(1-(2-oxo-2*H*-chromen-3-yl)ethylidene)-acetohydrazide (**12**) with substituted benzaldehydes gave the respective arylidene derivatives **13a**–**c** under the conditions employed. The structures of the synthesized compounds were assigned based on elemental analyses and spectral data. Also, the cytototoxic activities of the thiazole derivative **5a** was evaluated against HaCaT cells (human keratinocytes). It was found that compound **5a** possess potent cytotoxic activity.

## 1. Introduction

The synthesis of coumarins and their derivatives has attracted considerable attention from organic and medicinal chemists for many years as a large number of natural and synthetic products contain this heterocyclic nucleus [1,2,3,4,5]. A number of natural and synthetic coumarin derivatives have been reported to exert notable antimicrobial [1,2], antifungal [3,4] and cytotoxic [5] activity.

Thiazole derivatives have also attracted increasing attention due to their numerous pharmacological applications and biological activities, such as anti-inflammatory, analgesic, antimicrobial, anti-HIV, antihypertensive and herbicidal activity [6,7,8,9,10].

Ultrasonic-assisted organic synthesis (UAOS) is a powerful and green approach which is being used more and more to accelerate synthesis of organic compounds [11]. Increases in reaction rate and yields occur on application of ultrasound waves [12,13,14,15,16].

In view of these observations and in continuation of our previous work on the synthesis of heterocyclic systems for biological evaluation [17,18,19], we report herein a facile route to various thiazole derivatives incorporating coumarin moieties using the ultra-sound irradiation technique. Additionally we have found that one of the synthesized compounds has shown high cytotoxic activity.

## 2. Results and Discussion

### 2.1. Chemistry

**Scheme 1:**
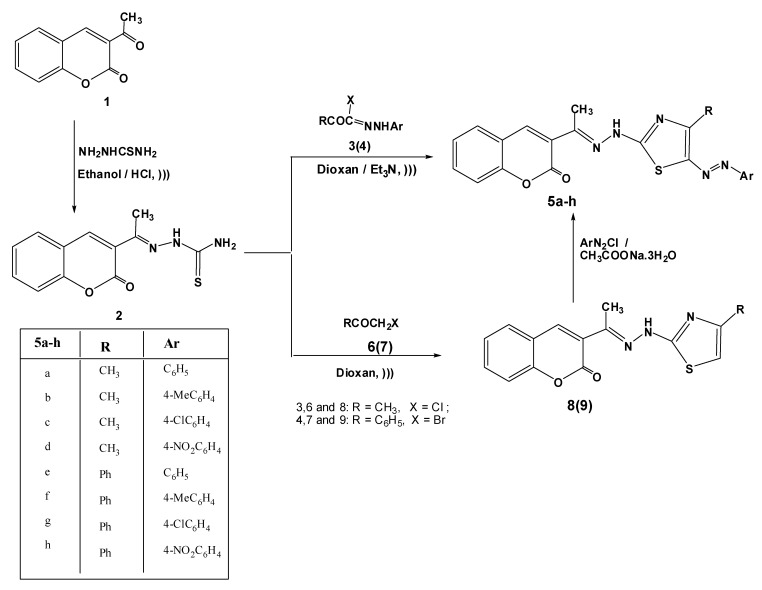
Synthesis of 5-arylazothiazole derivatives **5a**–**h**.

2-(1-(2-Oxo-2*H*-chromen-3-yl)ethylidene)thiosemicarbazide (**2**) was previously prepared by refluxing 3-acetyl-2*H*-chromen-2-one (**1**) and thiosemicarbazide in absolute ethanol in the presence of catalytic amounts of HCl [20] (Scheme 1).

The target compounds **5a**–**h**, 3-[1-(4-substituted-5-(aryldiazenyl)thiazol-2-yl)hydrazono) ethyl]-2*H*-chromen-2-ones, were synthesized in a one pot reaction of thiosemicarbazide **2** and hydrazonoyl halides **3**(**4**) in the presence of TEA under ultrasonic irradiation (Scheme 1).

The structural elucidation of the compounds was based on spectral evidence and microanalyses. The mass spectra of these products **5a**–**c** showed the molecular ion peaks at the expected *m/z* values. Their IR spectra showed the disappearance of the NH_2_ group, and revealed in each case one band at 1568–1558 cm^−1^, assignable to the N=N group (see Experimental).

The thiazole derivatives **8**(**9**) were synthesized in good yields by the treatment of thiosemicarbazide derivative **2** with chloroacetone (**6**) or phenacyl bromide (**7**) in dioxane under ultrasonic irradiation following the Hantzsch thiazole synthesis [21]. Upon coupling the thiazole derivatives **8**(**9**) with diazotized aniline, in presence of sodium acetate trihydrate, the azo derivatives **5a**–**h** were obtained. The structures of the latter products were confirmed by the appearance of a N=N band in the IR spectra and the lack of signals due to the C-5 proton of the thiazole ring in their ^1^H-NMR spectra (see Experimental). The azo derivatives of similar thiazoles have found wide applications in the dyeing of synthetic fibers [22,23] and the azo derivatives described in the present work may find similar applications. 

4-Thiazolidinone compound **11** was obtained by reaction of thiosemicarbazide **2** with chloroacetic acid in glacial acetic acid and in the presence of anhydrous sodium acetate. Reaction of the latter product **11** with substituted aldehydes afforded the corresponding arylidines **10a**–**d**.

**Scheme 2:**
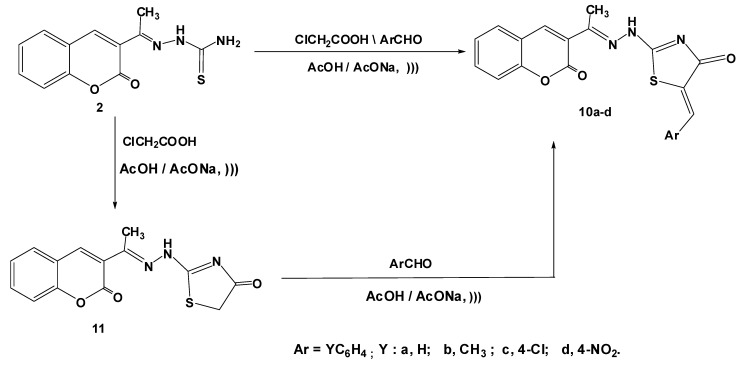
Synthesis of 5-arylidenethiazolinone derivatives **10a**–**d**.

The one pot synthesis of products **10a**–**d** has been carried out via reaction of thiosemicarbazide **2** with chloroacetic acid and aldehydes in glacial acetic acid in presence of excess anhydrous sodium acetate (Scheme 2). The ^1^H-NMR spectra data were also consistent with the assigned structures; thiazolidinone CH_2_ protons of **11** appeared at δ 3.97 ppm, arylidiene CH proton of **10a**–**d** was observed at 8.60–8.67 ppm (see Experimental).

**Scheme 3:**
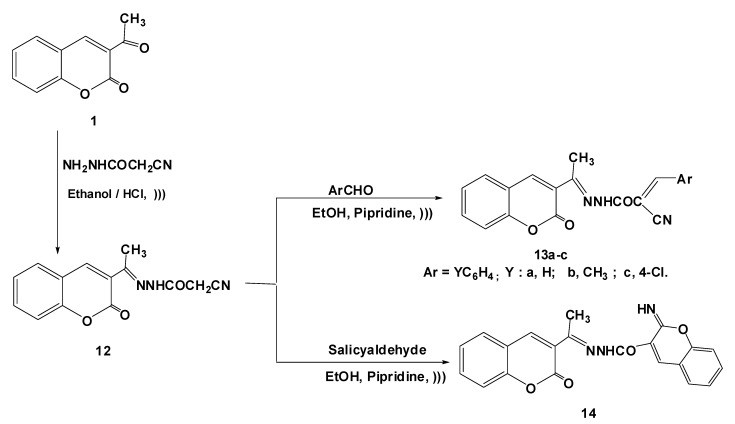
Reaction of acetohydrazide 12 with aromatic aldehydes.

In addition, the hydrazide-hydrazone derivative **12** was prepared by ultrasonic irradiation of 3-acetyl-2*H*-chromen-2-one (**1**) and 2-cyanoacetohydrazide in absolute ethanol in the presence of catalytic amounts of HCl (Scheme 3). The structure of compound **12** was established on the basis of analytical and spectral data. Thus its ^1^H-NMR spectrum showed the presence of a singlet at δ 4.25 ppm for the CH_2_ group, and a singlet at δ 11.19 ppm for an NH group. Its mass spectrum revealed a molecular ion peak at *m/z* 269 (see Experimental).

Furthermore, treatment of the acetohydrazide **12** with substituted benzaldehydes, under ultrasonic irradiation, afforded the benzylidene derivatives **13a**–**c** on the basis of their spectral data (Scheme 3) which confirmed the structures of the products by the appearance of a C=CH signal at δ 9.34 ppm and the lack of the characteristic signal due to methylene protons (see Experimental). The reaction of **12** with salicylaldehyde gave the coumarin derivative **14** (Scheme 3), in analogy with the reported literature [24,25]. The IR spectrum of compound **14** showed the lack of absorption bands corresponding to a C≡N group and presence of bands at 3,198 cm^−1^ due to the NH group.

### 2.2. *In Vitro* Cytotoxicity Assay

**Figure 1:**
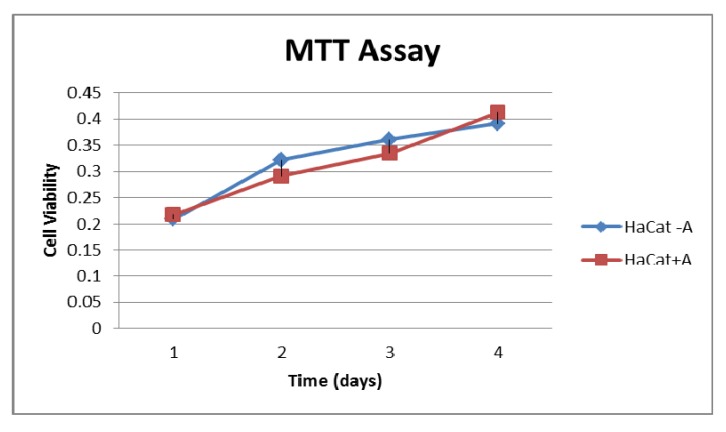
Represents the MTT assay results of healthy cells HaCat cells incubated with 50 μL 0.5 mol **5a** compared to control one. The used concentration of **5a** does not induce significant cytotoxic effect on the healthy HaCaT cells.

The effect of compound **5a** on cellular viability was studied using the MTT Assay. The HaCaT cells are plated and cultured in 12-well cell culture plates for 24 h (four plates represent the four days incubation with **5a**, each plate divided into 6 wells as control and 6 wells as a test) (Figure 1).

## 3. Experimental

### 3.1. Chemistry

#### 3.1.1. General

Melting points were measured on an Electrothermal IA 9000 series digital melting point apparatus. The IR spectra were recorded in potassium bromide discs on a Pye Unicam SP 3300 or a Shimadzu FT IR 8101 PC infrared spectrophotometers. The NMR Spectra were recorded at 300 MHz on a Varian Mercury VX-300 NMR spectrometer. ^1^H-NMR (300 MHz) and ^13^C-NMR (75 MHz) were run in deuterated dimethylsulphoxide (DMSO-*d*_6_). Chemical shifts were related to that of the solvent. Mass spectra were recorded on a Shimadzu GCMS-QP1000 EX mass spectrometer at 70 eV. Elemental analyses of the products were carried out at the Microanalytical Centre of Cairo University, Giza, Egypt. All reactions were followed by TLC (Silica gel, Merck). Irradiation was done in an ultrasonicator, (Electric supply: 230 v, A.C. 50 Hz, 1phase; Ultrasonic frequency: 36 KHz; Ultrasonic power: 100 W). *In vitro* cytotoxicity assay was performed at Regional Center for Food & Feed, Agricultural Research Center, Giza, Egypt, using the MTT Assay. 2-(1-(2-oxo-2*H*-chromen-3-yl)ethylidene)thiosemicarbazide (**2**) [20] and hydrazonoyl halides **3** [26,27] were prepared as reported in the literature.

#### 3.1.2. Synthesis of 3-[1-(4-Substituted-5-(aryldiazenyl)thiazol-2-yl)hydrazono) ethyl]-2*H*-chromen-2-ones **5a**–**h**

##### 3.1.2.1. Method A: General Procedure

A mixture of 2-(1-(2-oxo-2*H*-chromen-3-yl)ethylidene) thiosemicarbazide (**2**, 0.522 g, 2 mmol) and appropriate hydrazonoyl halides **3**(**4**) (2 mmol) in dioxane (30 mL) containing triethylamine (0.2 g, 2 mmol) was irradiated by an ultrasonic generator in a water-bath at 50–60 °C for 30 min. (monitored by TLC). The formed yellow precipitate was isolated by filtration, washed with ethanol, dried and recrystallized from dioxane to give compounds **5a**–**h**.

*3-[1-(2-(4-Methyl-5-(phenyldiazenyl)thiazol-2-yl)hydrazono)ethyl]-2H-chromen-2-one* (**5a**). Yield 72%; yellow solid; mp = 142 °C; IR (KBr): *v* 1559 (N=N), 1624 (C=N), 1724 (C=O), 3408 (NH) cm^−1^; ^1^H-NMR (DMSO-*d*_6_): *δ* 2.20 (s, 3H, CH_3_), 2.38 (s, 3H, CH_3_), 7.12–7.89 (m, 9H, ArH), 8.37 (s, 1H, Coumarin-H_4_), 10.60 (s, 1H, D_2_O exchangeable, NH); ^13^C-NMR (DMSO-*d*_6_): *δ* 14.61 (CH_3_), 9.52 (CH_3_), 112.15, 114.45, 120.22, 120.55, 121.85, 123.25, 124.33, 125.75, 127.16, 127.31, 129.73, 131.61, 133.73, 138.61 (Ar-C and Ar-CH), 153.13(C=N), 157.32(C=N), 167.51 (C=O); MS *m/z* (%):404 (M^+^ + 1, 7), 403 (M^+^, 100), 106 (75), 91 (82), 77 (27). Anal. Calcd for C_21_H_17_N_5_O_2_S (403.11): C, 62.52; H, 4.25; N, 17.36. Found C, 62.42; H, 4.18; N, 17.12%.

*3-[1-(2-(4-Methyl-5-(p-tolyldiazenyl)thiazol-2-yl)hydrazono)ethyl]-2H-chromen-2-one* (**5b**). Yield 74%; yellow solid; mp = 230 °C. IR (KBr): *v* 1558 (N=N), 1623 (C=N), 1724 (C=O), 3408 (NH) cm^−1^; ^1^H-NMR (DMSO-*d*_6_): *δ* 2.20 (s, 3H, CH_3_), 2.38 (s, 3H, CH_3_), 2.58 (s, 3H, CH_3_), 7.12–7.88 (m, 8H, ArH), 8.35 (s, 1H, coumarin-H_4_), 10.63 (s, 1H, D_2_O exchangeable, NH); MS *m/z* (%): 417 (M^+^, 4), 106 (100), 91 (82), 65 (27). Anal. Calcd for C_22_H_19_N_5_O_2_S (417.13): C, 63.29; H, 4.59; N, 16.78. Found C, 63.22; H, 4.47; N, 16.53%.

*3-[1-(2-(5-((4-Chlorophenyl)diazenyl)-4-methylthiazol-2-yl)hydrazono)ethyl]-2H-chromen-2-one* (**5c**). Yield 76%; yellow solid; mp = 188 °C; IR (KBr): *v* 1564 (N=N), 1626 (C=N), 1732 (C=O), 3410 (NH) cm^−1^; ^1^H-NMR (DMSO-*d*_6_): *δ* 2.20 (s, 3H, CH_3_), 2.38 (s, 3H, CH_3_), 7.10–7.98 (m, 8H, ArH), 8.38 (s, 1H, coumarin-H_4_), 10.66 (s, 1H, D_2_O exchangeable, NH); MS *m/z* (%): 438 (M^+^ + 1, 8), 437 (M^+^, 16), 127 (100), 73 (68). Anal. Calcd for C_21_H_16_ClN_5_O_2_S (437.07): C, 57.60; H, 3.68; N, 15.99. Found C, 57.51; H, 3.62; N, 15.71%.

*3-[1-(2-(4-Methyl-5-((4-nitrophenyl)diazenyl)thiazol-2-yl)hydrazono)ethyl]-2H-chromen-2-one* (**5d**). Yield 73%; yellow solid; mp = 195 °C; IR (KBr): *v* 1568 (N=N), 1629 (C=N), 1702(C=O), 3404 (NH) cm^−1^; ^1^H-NMR (DMSO-*d*_6_): *δ* 2.22 (s, 3H, CH_3_), 2.40 (s, 3H, CH_3_), 7.12–7.95 (m, 8H, ArH), 8.39 (s, 1H, coumarin-H_4_), 10.68 (s, 1H, D_2_O exchangeable, NH); MS *m/z* (%): 448 (M^+^, 13), 201(54), 127 (100), 82 (94), 67 (100). Anal. Calcd for C_21_H_16_N_6_O_4_S (448.10): C, 56.24; H, 3.60; N, 18.74. Found C, 56.14; H, 3.65; N, 18.42%.

*3-[1-(2-(4-Phenyl-5-(phenyldiazenyl)thiazol-2-yl)hydrazono)ethyl]-2H-chromen-2-one* (**5e**). Yield 72%; yellow solid; mp = 182 °C; IR (KBr): *v* 1558 (N=N), 1626 (C=N), 1744 (C=O), 3413 (NH) cm^−1^; ^1^H-NMR (DMSO-*d*_6_): *δ* 2.44 (s, 3H, CH_3_), 7.12–7.95 (m, 14H, ArH), 8.30 (s, 1H, coumarin-H_4_), 10.56 (s, 1H, D_2_O exchangeable, NH); ^13^C-NMR (DMSO-*d*_6_): *δ* 10.31 (CH_3_), 112.35, 115.35, 119.43, 119.55, 121.35, 123.29, 123.52, 124.26, 125.12, 125.65, 126.34, 127.22, 129.28, 130.29, 132.33, 132.29, 136.67, 138.34 (Ar-C and Ar-CH), 154.27 (C=N), 155.98 (C=N), 167.29 (C=O); MS *m/z* (%): 465 (M^+^, 10), 359 (100), 134 (83), 89 (60), 77 (31). Anal. Calcd for C_26_H_19_N_5_O_2_S (465.13): C, 67.08; H, 4.11; N, 15.04. Found C, 67.00; H, 4.02; N, 14.86%. 

*3-[1-(2-(4-Phenyl-5-(p-tolyldiazenyl)thiazol-2-yl)hydrazono)ethyl]-2H-chromen-2-one* (**5f**). Yield 72%; yellow solid; mp = 178 °C; IR (KBr): *v* 1562 (N=N), 1628 (C=N), 1743 (C=O), 3415 (NH) cm^−1^; ^1^H-NMR (DMSO-*d*_6_): *δ* 2.20 (s, 3H, CH_3_), 2.44 (s, 3H, CH_3_), 7.12–7.95 (m, 13H, ArH), 8.31 (s, 1H, coumarin-H_4_), 10.59 (s, 1H, D_2_O exchangeable, NH); MS *m/z* (%): 480(M^+^ + 1, 7), 479 (M^+^, 100), 134 (83), 89 (82), 77 (31). Anal. Calcd for C_27_H_21_N_5_O_2_S (479.14): C, 67.62; H, 4.41; N, 14.60. Found C, 67.42; H, 4.51; N, 14.54%.

*3-[1-(2-(5-((4-Chlorophenyl)diazenyl)-4-phenylthiazol-2-yl)hydrazono)ethyl]-2H-chromen-2-one* (**5g**). Yield 72%; yellow solid; mp = 187 °C; IR (KBr): *v* 1565 (N=N), 1626 (C=N), 1742 (C=O), 3412 (NH) cm^−1^; ^1^H-NMR (DMSO-*d*_6_): *δ* 2.44 (s, 3H, CH_3_), 7.02–7.98 (m, 13H, ArH), 8.34 (s, 1H, coumarin-H_4_), 10.65 (s, 1H, D_2_O exchangeable, NH; MS *m/z* (%): 500 (M^+^ + 1, 8), 499 (M^+^, 28), 359 (100), 134 (64), 77 (18). Anal. Calcd for C_26_H_18_ClN_5_O_2_S (499.09): C, 62.46; H, 3.63; N, 14.01. Found C, 62.44; H, 3.56; N, 13.91%.

*3-[1-(2-(5-((4-Nitrophenyl)diazenyl)-4-phenylthiazol-2-yl)hydrazono)ethyl]-2H-chromen-2-one* (**5h**). Yield 72%; brown solid; mp = 178 °C; IR (KBr): *v* 1566 (N=N), 1628 (C=N), 1742(C=O), 3414 (NH) cm^−1^; ^1^H-NMR (DMSO-*d*_6_): *δ* 2.28 (s, 3H, CH_3_), 7.12–7.95 (m, 13H, ArH), 8.34 (s, 1H, coumarin-H_4_), 10.69 (s, 1H, D_2_O exchangeable, NH); MS *m/z* (%): 511(M^+^ + 1, 14), 510 (M^+^, 46), 417 (65), 257 (24), 107 (1), 67 (100). Anal. Calcd for C_26_H_18_N_6_O_4_S (510.11): C, 61.17; H, 3.55; N, 16.46. Found C, 61.11; H, 3.48; N, 16.34%.

##### 3.1.2.2. Method B

###### 3.1.2.2.1. Synthesis of 3-[1-((4-Substituted thiazol-2-yl)hydrazono)ethyl]-2*H*-chromen-2-ones **8**,**9**

A mixture of **2** (0.27 g, 1 mmol) and chloroacetone (**6**) or phenacyl bromide (**7**) (1 mmol) in absolute ethanol (30 mL) was irradiated with an ultrasonic generator in a water-bath at 50–60 °C for 20 min. (monitored by TLC). The product started to separate out during the course of reaction. The crystalline solid was filtered, washed with water, dried and recrystallized from DMF to give the corresponding compounds **8** and **9**, respectively.

*3-[1-(4-Methylthiazol-2-yl)hydrazono)ethyl]-2H-chromen-2-one* (**8**). Yield 79%; yellow solid; mp = 170 °C; IR (KBr): *v* 1721 (C=O), 3267 (NH) cm^−1^; ^1^H-NMR (DMSO-*d*_6_): *δ* 2.24 (s, 3H, CH_3_), 2.51 (s, 3H, CH_3_), 7.07–8.02 (m, 5H, ArH and thiazole-H_5_), 8.22 (s, 1H, coumarin-H_4_), 9.31 (s, 1H, D_2_O exchangeable, NH); MS *m/z* (%): 299 (M^+^, 6), 129 (29), 77 (12), 60 (100). Anal. Calcd for C_15_H_13_N_3_O_2_S (299.07): C, 60.18; H, 4.38; N, 14.04. Found C, 60.10; H, 4.28; N, 13.84%.

*3-[1-((4-Phenylthiazol-2-yl)hydrazono)ethyl]-2H-chromen-2-one* (**9**). Yield 84%; yellow solid; mp = 232 °C; IR (KBr): *v* 1744 (C=O), 3418 (NH) cm^−1^; ^1^H-NMR (DMSO-*d*_6_): *δ* 2.44 (s, 3H, CH_3_), 7.10–8.02 (m, 10H, ArH and thiazole-H_5_), 8.26 (s, 1H, coumarin-H_4_), 9.28 (s, 1H, D_2_O exchangeable, NH); ^13^C-NMR (DMSO-*d*_6_): *δ* 10.11 (CH_3_), 112.11, 116.28, 119.42, 120.36, 122.29, 123.01, 124.33, 125.75, 127.16, 127.31, 129.73, 134.33, 135.64, 139.83 (Ar-C and Ar-CH), 154.24 (C=N), 158.12 (C=N), 167.21 (C=O); MS *m/z* (%): 362 (M^+^ + 1, 12), 361 (M^+^, 48), 359 (100), 133 (61), 89 (81) 77 (32). Anal. Calcd for C_20_H_15_N_3_O_2_S (361.09): C, 66.46; H, 4.18; N, 11.63. Found C, C, 66.53; H, 4.10; N, 11.43%.

###### 3.1.2.2.2. Coupling of **8**(**9**) with Arenediazonium Chlorides

To a solution of **8** or **9** (1 mmol) in ethanol (20 mL) was added sodium acetate trihydrate (0.138 g, 1 mmol), and the mixture was cooled to 0–5 °C in an ice bath. To the resulting cold solution was added portionwise a cold solution of arenediazonium chloride [prepared by diazotizing aniline derivatives (1 mmol) dissolved in hydrochloric acid (6 M, 1 mL) with a solution of sodium nitrite (0.07 g, 1 mmol) in water (2 mL)]. After complete addition of the diazonium salt, the reaction mixture was stirred for a further 30 min in an ice bath. The solid that separated was filtered off, washed with water and finally recrystallized from ethanol to give product proved to be identical in all respects (mp, mixed mp and IR spectra) with compounds **5a**–**h** which obtained from method A.

#### 3.1.3. Synthesis of 5-Arylidene-2-(2-(1-(2-oxo-2*H*-chromen-3-yl)ethylidene)hydrazinyl)thiazol-4(5*H*)-ones **10a**–**d**

##### 3.1.3.1. Method A

A mixture of **2** (0.261 g, 1 mmol), chloroacetic acid (0.1 g, 1 mmol) and appropriate aldehyde (1 mmol) in glacial acetic acid (20 mL) containing anhydrous sodium acetate (0.33 g, 4 mmol) was irradiated with an ultrasonic generator in a water-bath at 50–60 °C for 30 min. (monitored by TLC). The reaction mixture was left to cool and the formed solid was filtered off, washed with water, dried and recrystallized from ethanol to give **10a**–**d**.

*5-Benzylidene-2-[2-(1-(2-oxo-2H-chromen-3-yl)ethylidene)hydrazinyl]thiazol-4(5H)-one* (**10a**). Yield 84%; yellow solid; mp = 176 °C; IR (KBr): *v* 1678, 1725 (2C=O), 3360 (NH) cm^−1^; ^1^H-NMR (DMSO-*d*_6_): *δ* 2.24 (s, 3H, CH_3_), 6.91–7.88 (m, 9H, ArH), 8.19 (s, 1H, coumarin-H_4_), 8.63 (s, 1H, N=CH), 10.87 (s, 1H, D_2_O exchangeable, NH); ^13^C-NMR (DMSO-*d*_6_): *δ* 9.97 (CH_3_), 112.15, 114.45, 121.23, 121.62, 121.89, 122.42, 124.19, 124.84, 126.46, 128.43, 129.87, 130.41, 134.43, 146.73 (Ar-C and Ar-CH), 154.19 (C=N), 158.12 (C=N), 167.11 (C=O), 181.56 (C=O); MS *m/z* (%): 302 (M^+^ + 1, 9), 301 (M^+^, 62), 218 (65), 172 (40), 130 (100), 77 (43). Anal. Calcd for C_21_H_15_N_3_O_3_S (389.08): C, 64.77; H, 3.88; N, 10.79. Found C, 64.58; H, 3.78; N, 10.48%.

*5-[4-Methylbenzylidene)-2-(2-(1-(2-oxo-2H-chromen-3-yl)ethylidene)hydrazinyl)thiazol-4(5H)-one* (**10b**). Yield 79%; yellow solid; mp = 172 °C; IR (KBr): *v* 1678, 1721 (2C=O), 3353 (NH) cm^−1^;^1^H-NMR (DMSO-*d*_6_): *δ* 2.24 (s, 3H, CH_3_), 2.51 (s, 3H, CH_3_), 6.91–7.88 (m, 8H, ArH), 8.19 (s, 1H, coumarin-H_4_), 8.60 (s, 1H, N=CH), 10.83 (s, 1H, D_2_O exchangeable, NH); MS *m/z* (%): 404 (M^+^ + 1, 15), 403 (M^+^, 46), 235 (100), 146 (64), 130 (18), 77 (39). Anal. Calcd for C_22_H_17_N_3_O_3_S (403.10): C, 65.49; H, 4.25; N, 10.42. Found C, 65.43; H, 4.11; N, 10.12%.

*5-(4-Chlorobenzylidene)-2-[2-(1-(2-oxo-2H-chromen-3-yl)ethylidene)hydrazinyl]thiazol-4(5H)-one* (**10c**). Yield 82%; yellow solid; mp = 186 °C; IR (KBr): *v* 1679, 1722 (2C=O), 3353 (NH) cm^−1^; ^1^H-NMR (DMSO-*d*_6_): *δ* 2.26 (s, 3H, CH_3_), 6.91–7.96 (m, 8H, ArH), 8.19 (s, 1H, coumarin-H_4_), 8.62 (s, 1H, N=CH), 10.86 (s, 1H, D_2_O exchangeable, NH); MS *m/z* (%): 424 (M^+^ + 1, 13), 423 (M^+^, 46), 235 (100), 172 (36), 130 (58), 63 (69). Anal. Calcd for C_21_H_14_ClN_3_O_3_S (423.04): C, 59.50; H, 3.33; N, 9.91. Found C, 59.37; H, 3.13; N, 9.61%.

*5-(4-Nitrobenzylidene)-2-[2-(1-(2-oxo-2H-chromen-3-yl)ethylidene)hydrazinyl]thiazol-4(5H)-one* (**10d**). Yield 76%; yellow solid; mp = 170 °C; IR (KBr): *v* 1679, 1723 (2C=O), 3366 (NH) cm^−1^; ^1^H-NMR (DMSO-*d*_6_): *δ* 2.26 (s, 3H, CH_3_), 6.91–7.96 (m, 8H, ArH), 8.16 (s, 1H, coumarin-H_4_), 8.67 (s, 1H, N=CH), 10.86 (s, 1H, D_2_O exchangeable, NH); MS *m/z* (%): 435 (M^+^ + 1, 7), 434 (M^+^, 44), 235 (100), 218 (66), 130 (70), 63 (58). Anal. Calcd for C_21_H_14_N_4_O_5_S (434.07): C, 58.06; H, 3.25; N, 12.90. Found C, 57.86; H, 3.26; N, 12.79%.

##### 3.1.3.2. Method B

###### 3.1.3.2.1. Synthesis of 2-[2-(1-(2-oxo-2*H*-chromen-3-yl)ethylidene)hydrazinyl]thiazol-4(5*H*)-one (**11**)

A mixture of **2** (0.261 g, 1 mmol) and chloroacetic acid (0.1 g, 1 mmol) in glacial acetic acid (30 mL) containing anhydrous sodium acetate (0.33 g, 4 mmol) was irradiated with an ultrasonic generator in a water-bath at 50–60 °C for 30 min. (monitored by TLC). The reaction mixture was cooled and the resulting precipitate was filtered off and recrystallized from ethanol to give **11**. Yield 84%; yellow solid; mp = 220 °C; IR (KBr): *v* 1667, 1717 (2C=O), 3167 (NH) cm^−1^; ^1^H-NMR (DMSO-*d*_6_): *δ* 2.26 (s, 3H, CH_3_), 3.97 (s, 2H, CH_2_), 6.91–8.19 (m, 4H, ArH), 8.63 (s, 1H, coumarin-H_4_), 10.87 (s, 1H, D_2_O exchangeable, NH); ^13^C-NMR (DMSO-*d*_6_): *δ* 10.46 (CH_3_), 39.35 (CH_2_), 118.45, 120.18, 122.23, 124.33, 129.73, 131.61, 133.73, 138.61 (Ar-C and Ar-CH), 153.43 (C=N), 156.56 (C=N), 167.12 (C=O), 176.33 (C=O); MS *m/z* (%): 303 (M^+^ + 2, 3), 302 (M^+^ + 1, 9), 301 (M^+^, 36), 235 (73), 146 (36), 130 (100), 77 (59). Anal. Calcd for C_14_H_11_N_3_O_3_S (301.05): C, 55.80; H, 3.68; N, 13.95. Found C, 55.68; H, 3.60; N, 13.75%.

###### 3.1.3.2.2. Reaction of **8** with Aromatic Aldehydes

*General procedure:* To a solution of 5-thiazolidinone **11** (0.30 g, 1 mmol) and appropriate aldehyde (1 mmol) in glacial acetic acid (20 mL), anhydrous sodium acetate (0.33 g, 4 mmol) was irradiated with an ultrasonic generator in a water-bath at 50–60 °C for 30 min (monitored by TLC). The product, so separated, was filtered, washed with water, dried and recrystallized from ethanol to give compounds which proved to be identical in all respects (mp, mixed mp and IR spectra) with the hydrazonothiazolidinones **10a**–**d** which obtained from method A.

#### 3.1.4. Synthesis of 2-Cyano-*N'*-(1-(2-oxo-2*H*-chromen-3-yl)ethylidene)acetohydrazide (**12**)

To a solution of 2-cyanoacetohydrazide (1.0 g, 10 mmol) and 3-acetyl-2*H*-chromen-2-one (**1**, 1.88 g, 10 mmol) in absolute ethanol (30 mL) three drops of conc. HCl were added and the reaction mixture was irradiated with an ultrasonic generator in a water-bath at 50–60 °C for 20 min. then left to cool. The solid product formed was collected by filtration, dried and recrystallized from ethanol to give **12**. Yield 86%; yellow microcrystals; mp = 172 °C; IR (KBr): *v* = 1690, 1724 (2C=O), 2230 (CN), 3186 (NH) cm^−1^; ^1^H-NMR (DMSO-*d*_6_): *δ* 2.17 (s, 3H, CH_3_), 4.25 (s, 2H, CH_2_), 7.39–8.30 (m, 4H, ArH), 8.95 (s, 1H, coumarin-H_4_), 11.19 (s, 1H, D_2_O exchangeable, NH); MS *m/z* (%): 270 (M^+^ + 1, 5), 269 (M^+^, 33), 229 (82), 115 (100), 89 (60), 63 (54). Anal. Calcd for C_14_H_11_N_3_O_3_ (269.08): C, 62.45; H, 4.12; N, 15.61. Found C, C, 62.41; H, 4.02; N, 15.38%.

#### 3.1.5. Reaction of **11** with Aromatic Aldehydes

*General procedure:* Equimolecular mixture of 2-cyano-*N'*-(1-(2-oxo-2*H*-chromen-3-yl)ethylidene) acetohydrazide (**12**, 2.69 g, 0.01 mol) and appropriate aldehyde (0.01 mol), in anhydrous ethanol (20 mL) containing piperidine (0.50 mL) was irradiated with an ultrasonic generator in a water-bath at 50–60 °C for 30 min (monitored by TLC). The formed solid was collected by filtration and recrystallized from the proper solvent to give compounds **13a**–**d**.

*2-Cyano-N'-[1-(2-oxo-2H-chromen-3-yl)ethylidene]-3-phenylacrylohydrazide* (**13a**). Yield 80%; yellow solid (from ethanol); mp = 198 °C; IR (KBr): *v* 1672, 1728 (2C=O), 2210 (CN), 3336 (NH) cm^−1^; ^1^H-NMR (DMSO-*d*_6_): *δ* 2.19 (s, 3H, CH_3_), 7.24–8.21(m, 9H, ArH ), 8.87 (s, 1H, coumarin-H_4_), 9.34 (s, 1H, C=CH), 11.22 (s, 1H, D_2_O exchangeable, NH); ^13^C-NMR (DMSO-*d*_6_): *δ* 10.74 (CH_3_), 112.36, 115.26, 117.26, 119.42, 121.65, 122.86, 123.43, 124.16, 124.86, 127.67, 127.99, 129.04, 133.43, 134.87, 139.37 (Ar-C, Ar-CH and C≡N), 154.94 (C=N), 168.13 (C=O), 174.14 (C=O); MS *m/z* (%): 358 (M^+^ + 1, 4), 357 (M^+^, 40), 185 (100), 129 (73), 109 (73), 55 (91). Anal. Calcd for C_21_H_15_N_3_O_3_ (357.11): C, 70.58; H, 4.23; N, 11.76. Found C, 70.49; H, 4.21; N, 11.46%.

*2-Cyano-N'-[1-(2-oxo-2H-chromen-3-yl)ethylidene]-3-p-tolylacrylohydrazide* (**13b**). Yield 82%; yellow solid (from DMF); mp = 187 °C. IR (KBr): *v* 1672, 1728 (2C=O), 2216 (CN), 3336 (NH) cm^−1^; ^1^H-NMR (DMSO-*d*_6_): *δ* 2.19 (s, 3H, CH_3_), 2.49 (s, 3H, CH_3_), 7.20–8.23 (m, 8H, ArH), 8.89 (s, 1H, coumarin-H_4_), 9.34 (s, 1H, C=CH), 11.26 (s, 1H, D_2_O exchangeable, NH); MS *m/z* (%): 372 (M^+^ + 1, 6), 371 (M^+^, 45), 229 (75), 186 (55), 115 (100), 63 (62). Anal. Calcd for C_22_H_17_N_3_O_3_(371.13): C, 71.15; H, 4.61; N, 11.31. Found C, C, 71.05; H, 4.36; N, 11.11%. 

*3-(4-Chlorophenyl)-2-cyano-N'-(1-(2-oxo-2H-chromen-3-yl)ethylidene)acrylohydrazide* (**13c**). Yield 78%; yellow solid (from ethanol ); mp = 202 °C; IR (KBr): *v* 1674, 1732 (2C=O), 2218 (CN), 3334 (NH) cm^−1^; ^1^H-NMR (DMSO-*d*_6_): *δ* 2.19 (s, 3H, CH_3_), 7.21–8.23(m, 8H, ArH), 8.89 (s, 1H, coumarin-H_4_), 9.35 (s, 1H, C=CH), 11.34 (s, 1H, D_2_O exchangeable, NH); MS *m/z* (%): 392 (M^+^ + 1, 7), 391 (M^+^, 22), 273 (47), 219 (100), 84(94). Anal. Calcd for C_21_H_14_ClN_3_O_3_ (391.07): C, 64.37; H, 3.60; N, 10.72. Found C, C, 64.07; H, 3.62; N, 10.32%.

*2-Imino-N'-[1-(2-oxo-2H-chromen-3-yl)ethylidene]-2H-chromene-3-carbohydrazide* (**14**). Yield 78%; yellow solid (from DMF); mp = 240 °C; IR (KBr): *v* 1669, 1712 (2C=O), 3198, 3325 (2NH) cm^−1^; ^1^H-NMR (DMSO-*d*_6_): *δ* 2.19 (s, 3H, CH_3_), 7.10–8.36 (m, 9H, ArH ), 8.44 (s, 1H, D_2_O exchangeable, NH), 8.89 (s, 1H, coumarin-H_4_), 11.38 (s, 1H, D_2_O exchangeable, NH); MS *m/z* (%): 374 (M^+^ + 1, 13), 373 (M^+^, 13), 201 (51), 171 (100), 115 (89), 62 (58). Anal. Calcd for C_21_H_15_N_3_O_4_ (373.11): C, 67.56; H, 4.05; N, 11.25. Found C, C, 67.44; H, 4.15; N, 11.05%. 

### 3.2. Cytotoxic Activity

The method applied is similar to that reported by Skehan *et al.* using the 3-(4,5-dimethylthiazol-2-yl)-2,5-diphenyl-2*H*-tetrazolium bromide (MTT) assay. Healthy HaCaT epithelial primary cell line (human keratinocytes) was cultured on 18 mm diameter glass cover slips in a 12-well tissue culture plate in DMEM plus 5% FBS at 37–38 °C under 5% CO_2_. The cover slips were coated with collagen type I (Roche) in advance for optimum cell growth. The HaCaT cells are cultured in 12-well cell culture plates for 24 h, (four plates represent the four days incubation with **5a**, each plate divided into 6-wells as control and 6-wells as a test). Rinsing the old medium and adding new one then 50 μL 0.5 mol **5a** are added to test wells (not to the control one) and returned to incubator for 8 h washing the excess **5a** by DPBS buffer and add 1 mL medium to each well then incubated for the designated periods for each plate (Figure 1).

## 4. Conclusions

In summary, we have developed a new green methodology and synthesized several 3-[1-(4-substituted-5-(aryldiazenyl)thiazol-2-yl)hydrazono)ethyl]-2*H*-chromen-2-ones by ultrasound irradiation. The cytototoxic activity of one of them was also evaluated against healthy HaCaT cells.
